# Improved efficacy of pembrolizumab combined with soluble EphB4-albumin in HPV-negative EphrinB2 positive head neck squamous cell carcinoma

**DOI:** 10.18632/oncotarget.28605

**Published:** 2024-07-10

**Authors:** Alexandra Jackovich, Barbara J. Gitlitz, Justin Wayne Wong Tiu-lim, Vinay Duddalwar, Kevin George King, Anthony B. El-Khoueiry, Jacob Stephen Thomas, Denice Tsao-Wei, David I. Quinn, Parkash S. Gill, Jorge J. Nieva

**Affiliations:** ^1^Rutgers New Jersey Medical School, Newark, NJ 07103, USA; ^2^Division of Medical Oncology, University of Southern California, Los Angeles, CA 90007, USA

**Keywords:** EphrinB2, EphB4, HNSCC, pembrolizumab, HPV-negative

## Abstract

Objective: Patients with relapsed or metastatic head and neck squamous cell carcinoma (HNSCC) after primary local therapy have low response rates with cetuximab, systemic chemotherapy or check point inhibitor therapy. Novel combination therapies with the potential to improve outcomes for patients with HNSCC is an area of high unmet need.

Methods: This is a phase II single-arm clinical trial of locally advanced or metastatic HNSCC patients treated with a combination of soluble EphB4-human serum albumin (sEphB4-HSA) fusion protein and pembrolizumab after platinum-based chemotherapy with up to 2 prior lines of treatment. The primary endpoints were safety and tolerability and the primary efficacy endpoint was overall response rate (ORR). Secondary endpoints included progression free survival (PFS) and overall survival (OS). HPV status and EphrinB2 expression were evaluated for outcome.

Results: Twenty-five patients were enrolled. Median follow up was 40.4 months (range 9.8 – 40.4). There were 6 responders (ORR 24%). There were 5 responders in the 11 HPV-negative and EphrinB2 positive patients, (ORR 45%) with 2 of these patients achieving a complete response (CR). The median PFS in HPV-negative/EphrinB2 positive patients was 3.2 months (95% CI 1.1, 7.3). Median OS in HPV-negative/EphrinB2 positive patients was 10.9 months (95% CI 2.0, 13.7). Hypertension, transaminitis and fatigue were the most common toxicities.

Discussion: The combination of sEphB4-HSA and pembrolizumab has a favorable toxicity profile and favorable activity particularly among HPV-negative EphrinB2 positive patients with HNSCC.

## INTRODUCTION

Head and neck squamous cell carcinoma (HNSCC) is the seventh most common cancer worldwide, with approximately 930,000 new cases and 470,000 deaths occurring globally in 2020 [1]. In the United States, an estimated 66,000 new cases and 15,000 deaths are attributable to HNSCC [2]. Major risk factors for HNSCC include tobacco smoking, alcohol consumption, and human papillomavirus (HPV) infection [3, 4]. Despite the decline in prevalence of tobacco use, it remains a primary contributing factor to development of non-HPV-related HNSCC in the United States. In recent years, this subset of patients continues to see worse outcomes than HPV-associated HNSCC patients with 2-year overall survival of 62% as compared with 95% for non-HPV-associated disease [5, 6].

For advanced and recurrent or refractory HNSCC, common treatment regimens after progression from 1st or 2nd line chemotherapy include targeted therapy or immunotherapy with a PD-1 or PD-L1 inhibitor, regardless of HPV status. Pembrolizumab is approved by the US Food and Drug Administration as 1st and 2nd line monotherapy and in combination with chemotherapy for relapsed and metastatic HNSCC and as monotherapy in the 2nd line. PD-1/PD-L1 inhibitors provide superior outcomes including tumor response in advanced or recurrent HNSCC when compared to traditional chemotherapy [7, 8]. In the KEYNOTE-055 trial of pembrolizumab for platinum and cetuximab refractory HNSCC, overall response rate was 16%, similar to HPV negative population (ORR 15%, 95% CI 10-23%), with progression free survival of 2.1 months and overall survival of 8 months. In KEYNOTE-012, without mandate for prior therapy, ORR was 18% for all patients, while in the KEYNOTE-048 trial as first-line therapy the overall response rate was 20% but with a lower response rate of 14% in HPV negative HNSCC [9, 10]. Patients with HNSCC who progressed during or after platinum-containing treatment, overall survival with pembrolizumab compared with docetaxel, methotrexate or cetuximab was 8.4 months vs. 6.9 months, respectively [7, 9, 10–12]. This observation was confirmed in a pooled analysis of checkpoint inhibitor trials with response rates for HPV-positive and HPV negative patients being 21.9% vs. 14.1%, respectively [13–16].

sEphB4-HSA is a human fusion protein consisting of EphB4 extracellular domain (sEphB4) and full length human serum albumin (HSA). sEphB4-HSA binds exclusively to EphrinB2, therefore blocking binding of endogenous EphB4-receptor and blocks bidirectional signaling [17, 18]. Type one receptor tyrosine kinase EphB4 and its trans-membrane ligand EphrinB2, normally expressed on venous and arterial endothelial cells, respectively, induce bidirectional signaling; forward-signaling in EphB4-receptor expressing cells, and reverse-signaling in EphrinB2-ligand expressing cells. This interaction is critically required for maturation of the developing vascular system in the embryo [19]. The EphB4-EphrinB2 interaction between tumor cell-tumor vessel also promotes tumor growth and enhances tumor angiogenesis, and prohibits immune cell trafficking into the tumor [20–22]. Forward signaling activates Ras-MAPK and PI3K pathway and reverse signaling activates Src mediated events [23]. EphB4 has autonomous function in the tumor cell to promote tumor cell proliferation and survival. Notably, EphB4 and EphrinB2 are highly expressed in head and neck cancers [24–27] and predict poor survival [17, 21, 28].

## RESULTS

### Patients

**Figure 1:**
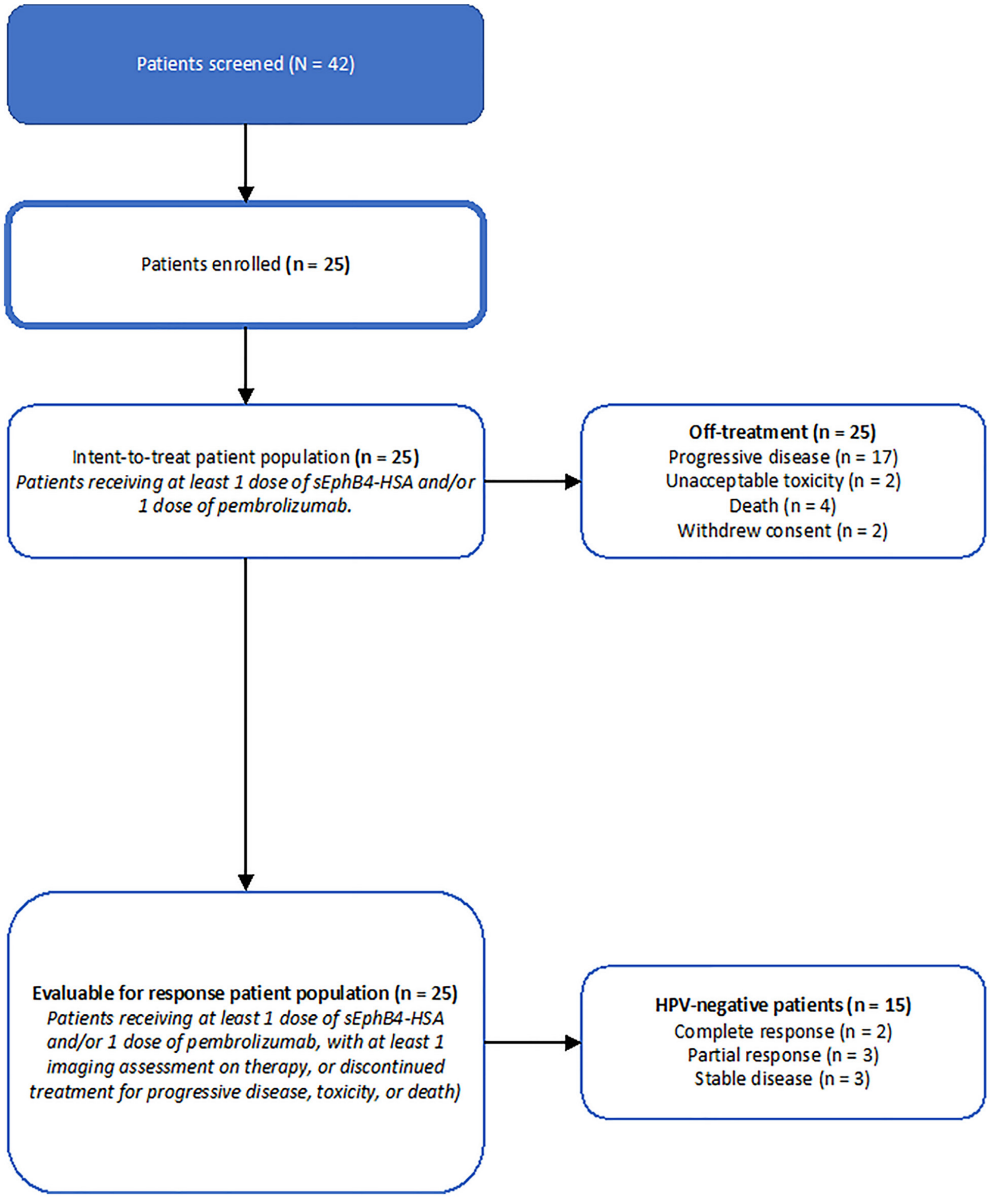
Consort diagram, clinical trial of sEphB4-HSA plus pembrolizumab.

Between June 21st, 2017, and February 22nd, 2021, 25 patients were enrolled. All patients received at least 1 dose of sEphB4-HSA and pembrolizumab. Therefore 25 patients were included in the analysis of safety. Median duration of follow up was 40.4 months (range 9.8–40.4). Three patients were not evaluable by radiographic response due to early discontinuation and no imaging study on therapy but were included in the intent-to-treat analysis (Figure 1). Fifteen (60%) patients were HPV-negative, 11 of whom were EphrinB2 positive. Patient demographics are shown in Table 1. The median age was 61 (range 31–79) years. Twenty (80%) patients were male (Table 1).

**Table 1:** Demographics of all patients and HPV negative, EphrinB2 positive patients

Characteristic	All accrued patients (*N* = 25)	HPV-Negative/EphrinB2-Positive patients (*n* = 11)
Age		
Median (range) — yr.	61 (31 — 79)	61 (38–78)
≥75 yr. — no. (%)	3 (12)	2 (18)
Male sex — no. (%)	20 (80)	8 (73)
Race/Ethnicity — no. (%)		
White	12 (48)	5 (45)
Hispanic^*^	6 (24)	3 (27)
Asian	5 (20)	3 (27)
Black	2 (8)	0
Smoking or tobacco use — no. (%)		
Current or former	13 (52)	8 (73)
Never	12 (48)	3 (27)
ECOG performance-status score — no. (%)		
0	12 (48)	5 (45)
1	13 (52)	6 (50)
Site of primary tumor — no. (%)		
Larynx	2 (8)	2 (18)
Oral cavity	10 (40)	4 (36)
Pharynx		
Nasopharynx	3 (12)	2 (18)
Oropharynx	6 (24)	0
Hypopharynx	1 (4)	1 (9)
Paranasal sinuses and nasal cavity	3 (12)	2 (18)
Sites of metastatic disease — no. (%)		
Lung	15 (60)	5 (45)
Liver	5 (20)	2 (18)
Bone	5 (20)	2 (18)
Adrenal Gland	2 (8)	0
Node Only	7 (28)	5 (45)
No. of prior regimens received for systemic cancer therapy — no. (%)		
0	5 (20)	1 (9)
1	18 (72)	8 (73)
2	2 (8)	2 (18)
Context of previous systemic therapy regimen — no. (%)		
Adjuvant therapy	6 (24)	3 (27)
Neoadjuvant therapy	3 (12)	1 (9)
Primary disease	7 (28)	3 (27)
Disease recurrence	2 (8)	0
Metastatic disease	2 (8)	3 (27)
No previous systemic therapy	5 (20)	1 (9)
Previous receipt of cetuximab — no. (%)	1 (4)	1 (9)

^*^Including all patients whose ethnicity is Hispanic.

### Safety

The median number of 3-week cycles given was 6 (range 1–30) (Table 1). Sixteen (64%) of 25 patients experienced hypertension, which was the most frequently treatment-related adverse event (AE). Other commonly experienced AEs included fatigue (*n* = 4, 16%) and elevated ALT/AST (*n* = 3, 12%). Grade 3 toxicities were experienced by 12 patients. The most common grade 3 AEs observed were hypertension (*n* = 9, 36%), elevated ALT/AST, hyponatremia, and dysphagia (*n* = 2 in each, or 8% each). There were no grade 4 treatment-related AEs observed and no treatment-related deaths (Table 2). Two (8%) patients had dose modifications while on therapy and 8 (32%) patients had one or more dose interruptions due to toxicity. Grade 3 toxicities included dysphagia, anorexia, and hypoalbuminemia and were each reported in two (8%) patients; one patient had grade 3 anemia (4%). Dyspnea, generalized muscle weakness, hyperbilirubinemia, and elevated alkaline phosphatase were each reported in one (4%) patient (Table 2).

**Table 2:** Adverse events

Event	sEphB4-HSA + Pembrolizumab (*N* = 25)	
	Any grade No. (%)	Grade 3 or 4 No. (%)
Patients with ≥1 event	19 (76)	12 (48)
Hypothyroidism	1 (4)	0
Immune thrombocytopenic purpura	0	0
Abdominal pain	0	0
Colitis	0	0
Dysphagia	2 (8)	2 (8)
Nausea	1 (4)	0
Stomatitis	0	0
Facial edema	0	0
Fatigue	4 (16)	1 (4)
Hypertension	16 (64)	9 (36)
Dyspnea	1 (4)	1 (4)
Pneumonitis	0	0
Hyperglycemia	1 (4)	0
Dehydration	0	0
Decreased appetite	0	0
General muscle weakness	1 (4)	1 (4)
Elevated ALT/AST	3 (12)	2 (8)
Elevated ALP	1 (4)	1 (4)
Hyponatremia	2 (8)	2 (8)
Anemia	1 (4)	1 (4)
Hyperbilirubinemia	1 (4)	1 (4)

### Efficacy

**Figure 2:**
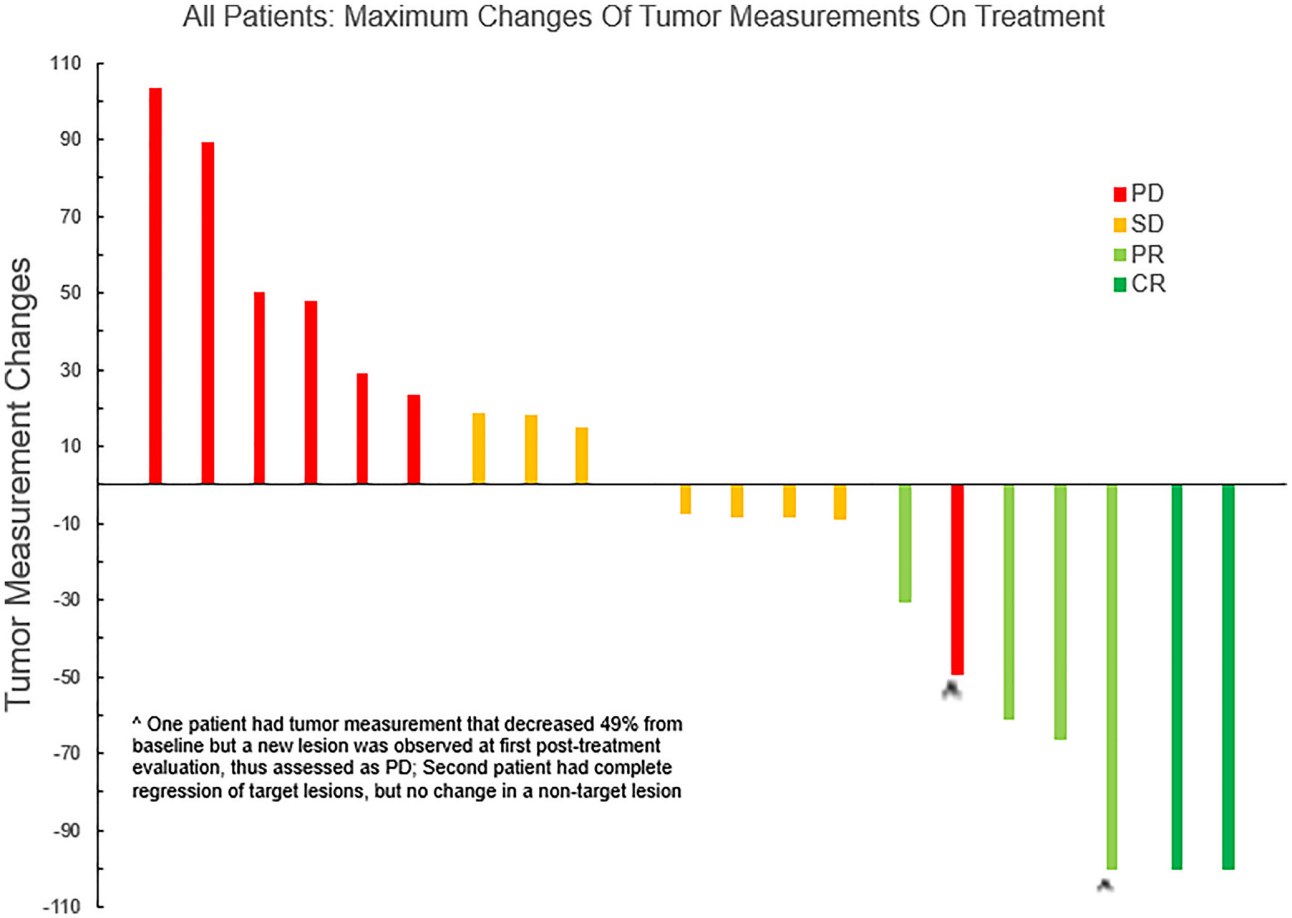
Waterfall plot, all patients with response assessed by imaging, clinical trial of sEphB4-HSA plus pembrolizumab (*n* = 21). Four patients did not have tumor assessment due to early withdrawal.

Overall response rate (ORR) in the intent-to-treat population of 25 patients was 24% (Table 3). Two (8%) of 25 patients had complete response (CR), 4 (16%) had partial response (PR), and 9 (36%) had stable disease (SD). (Table 3). The total disease control rate, which includes patients who experienced CR, PR, and SD for at least 6 months, was 7 (28%) out of 25 patients. There was one case of pseudo-progression, where subsequent imaging assessments on treatment demonstrated decrease in non-target lesions, while a target lesion in bone ultimately became sclerotic; this patient was not included in the ORR. Three (12%) of 25 patients with stable disease had no viable tumor on pathologic analysis on the week-8 repeat biopsy on therapy but were not included among responders. One of these patients with pathologic complete response (pCR) continued on treatment with durable disease control for 21 months. A waterfall plot shows the greatest percent tumor decreases for individual evaluable patients, defined as those with at least 1 imaging assessment on study (Figure 2).

**Table 3:** Efficacy of intent-to-treat population in all study patients and HPV-negative/EphrinB2 positive patients

	All Patients (*N* = 25)	HPV-Negative/EphrinB2-Positive patients (*n* = 11)
Overall Response Rate – no. (%) 95% CI	6 (24) (11%, 44%)	5 (45) (14%, 61%)
CR	2 (8)	2 (18)
PR	4 (16)	3 (27)
SD	9 (36)	3 (27)
PD	10 (40)	3 (27)
Overall Survival (Months) (95% CI)	8.6 (3.2, 13.7)	10.9 (2.0, 13.7)
Progression Free Survival (Months) (95% CI)	2.6 (1.3, 4.1)	3.2 (1.1, 7.3)

**Figure 3:**
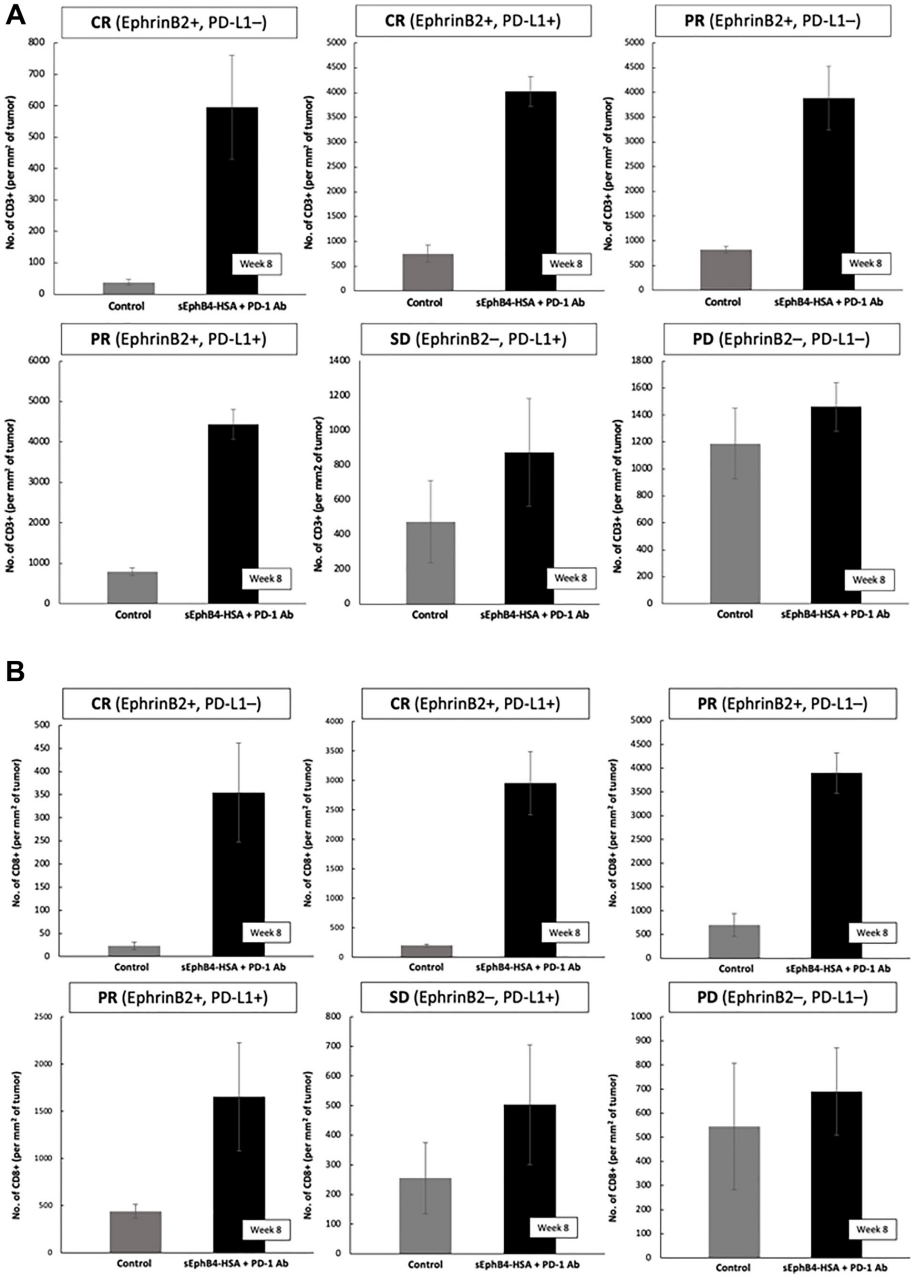
(**A**) Intatumoral CD8+ cell counts before initiation of therapy with sEphB4-HSA + pembrolizumab and on-therapy at week 8 biopsy. (**B**) Intatumoral CD8+ cell counts before initiation of therapy with sEphB4-HSA + pembrolizumab and on-therapy at week 8 biopsy.

In the HPV-negative group, radiographic response occurred in 5 of 15 (33%) patients, and all responders were EphrinB2 positive. There were 11 EphrinB2 positive/HPV-negative patients, who had an overall response rate of 45% (5 of 11). Two of these patients had complete remission. (Table 3). Rapid radiographic response was observed in 3 (27%) of 11 EphrinB2 positive/HPV-negative subgroup. Specifically, on CT-imaging assessments in these 3 patients, the measurable disease at baseline demonstrated tumor cavitation and rapid regression at 1st and 2nd CT-scan on therapy. A waterfall plot shows the greatest percent tumor decreases for individual evaluable HPV-negative patients, defined as those with at least 1 imaging assessment on study (Figure 3).

**Figure 4:**
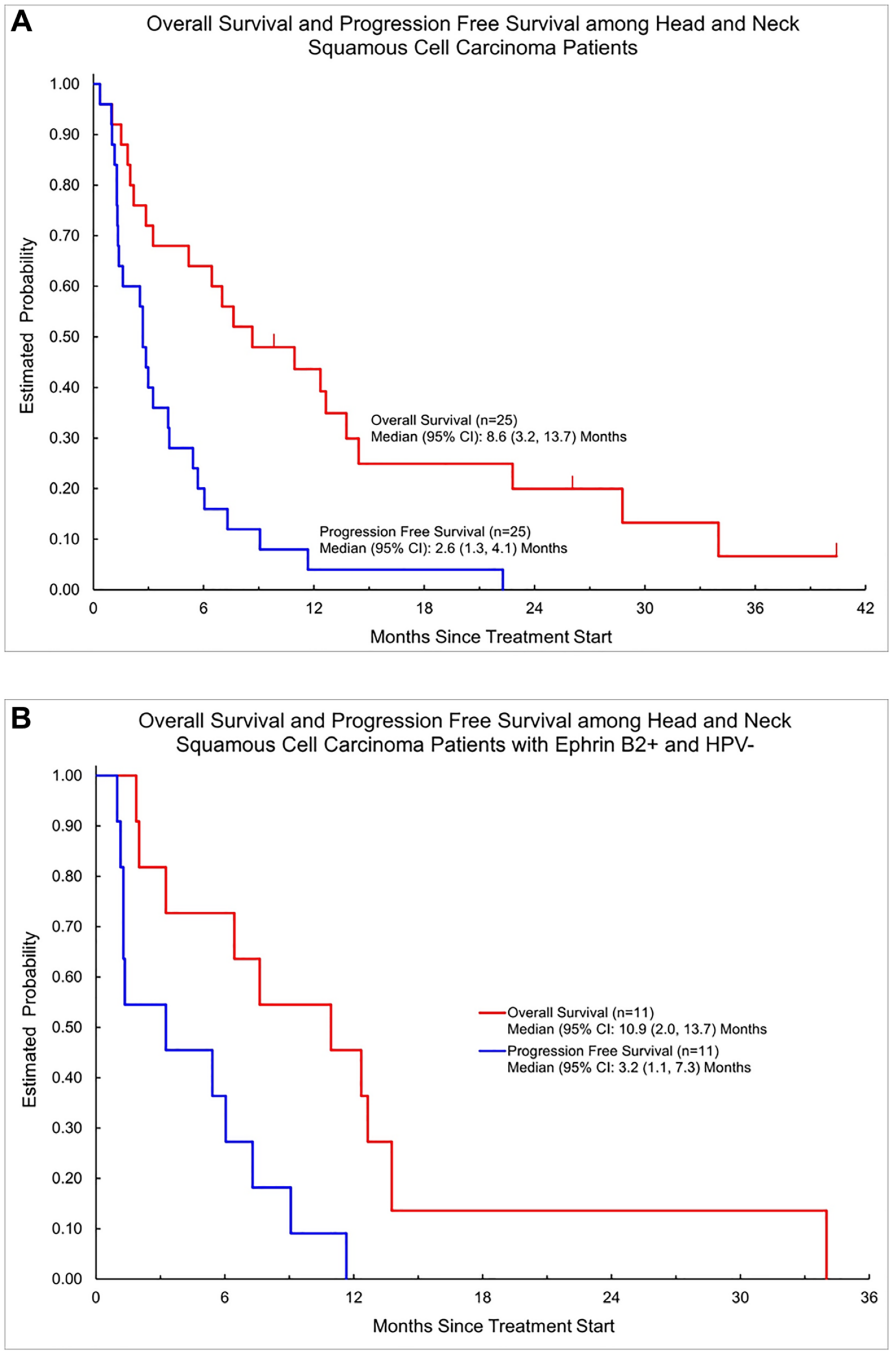
(**A**) Kaplan-Meier survival curve of all patients on clinical trial of sEphB4-HSA plus pembrolizumab. (**B**) Kaplan-Meier survival curve of HPV-negative patients on clinical trial of sEphB4-HSA plus pembrolizumab.

**Figure 5:**
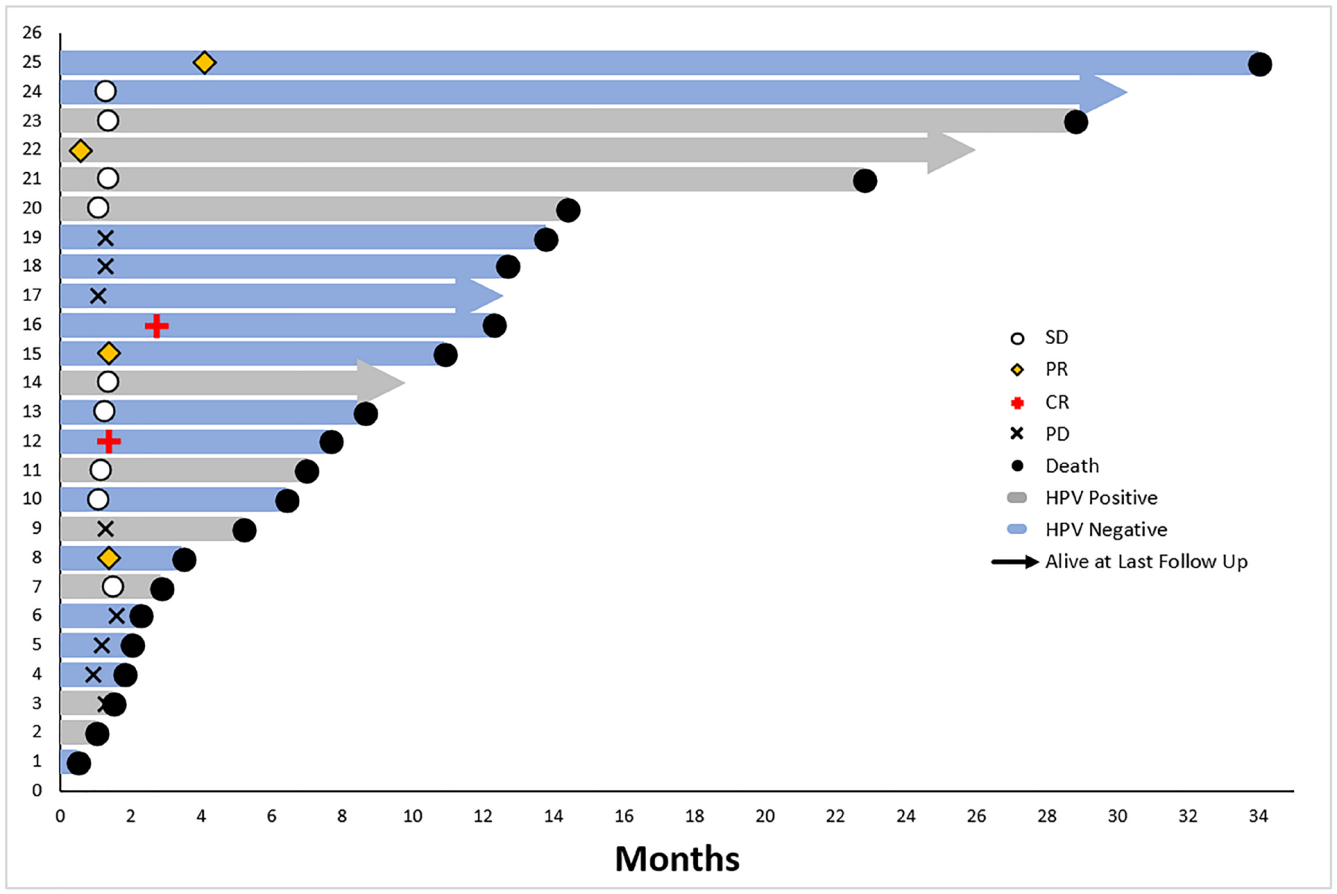
Overall survival swimmer plot, intent-to-treat patient population on clinical trial of sEphB4-HSA plus pembrolizumab (*n* = 25).

**Figure 6:**
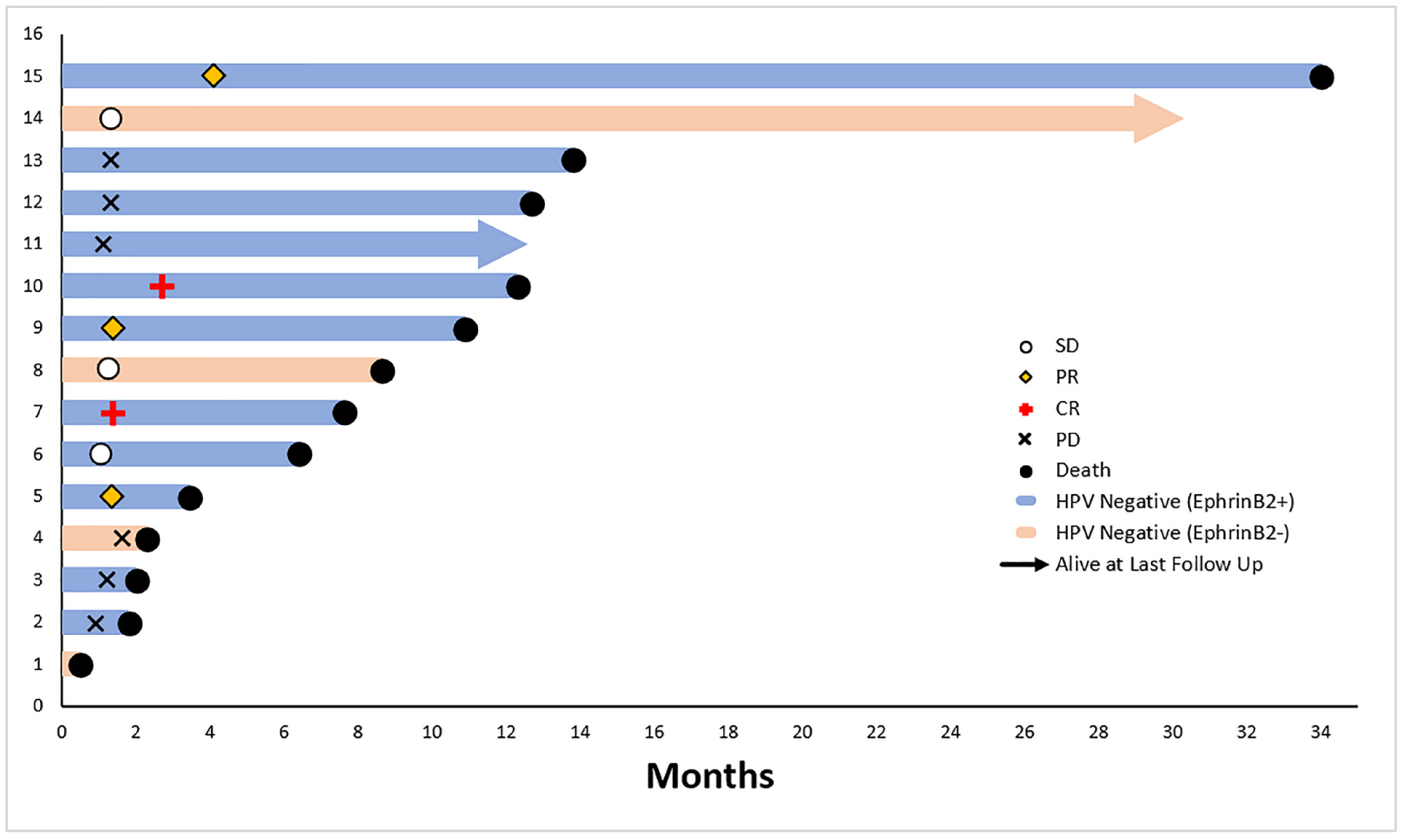
Overall survival swimmer plot, HPV-negative intent-to-treat patient population on clinical trial of sEphB4-HSA plus pembrolizumab (*n* = 15).

Median overall survival (OS) was 8.6 (95% CI: 3.2, 13.7) months in the total population of 25 patients. Median progression-free survival (PFS) was 2.6 (95% CI: 1.3, 4.1) months in the total population (Figure 4A). A swimmer plot shows individual patient overall survival and time of response among all patients in the intent-to-treat population (*N* = 25) (Figure 5). In the HPV-negative/EphrinB2 positive group, median OS was 10.9 (95% CI: 2.0, 13.7) months and median PFS was 3.2 (95% CI: 1.1, 7.3) months (Table 3, Figure 4B). A swimmer plot shows individual patient overall survival and time of response in the intent-to treat HPV-negative population (*n* = 15) (Figure 6).

### Biomarker analysis and immune cell infiltration analysis

EphrinB2 was expressed in 16 of 25 (64%) patients, which was more common in the HPV negative group Table 4. All 6 responding patients were among the EphrinB2 positive cases, 5 of whom were HPV negative and EphrinB2 positive. PD-L1 was positive in twelve (48%) of 25 patients. Three of the responding patients had both PD-L1 and EphrinB2 expression (Table 5).

**Table 4:** EphrinB2, PD-L1 expression and overall response rates in all patients (intent-to-treat population, *N* = 25)

Population (*N* = 25) ORR (%)	PD-L1 Positive (*n* = 12)	PD-L1 Negative (*n* = 13)	
**EphrinB2 positive** (*n* = 16)	3/8 (38)	3/8 (38)	*All EphrinB2 Pos.* 6/16 (37.5)
**EphrinB2 negative** (*n* = 9)	0/4 (0)	0/5 (0)	*All EphrinB2 Neg.* 0/9 (0)
	*All PD-L1 Pos.* 3/12 (25)	*All PD-L1 Neg.* 3/13 (23)	

**Table 5:** EphrinB2, PD-L1 expression and overall response rates in HPV-negative patients (intent-to-treat population, *n* = 15)

Population (*n* = 15) ORR (%)	PD-L1 Positive (*n* = 7)	PD-L1 Negative (*n* = 8)	
**EphrinB2 positive** (*n* = 11)	2/5 (40)	3/7 (60)	*All HPV Neg./EphrinB2 Pos.* 5/11 (45)
**EphrinB2 negative** (*n* = 4)	0/2 (0)	0/1 (0)	*All HPV Neg./EphrinB2 Neg.* 0/4 (0)
	*All HPV Neg./PD-L1 Pos.* 2/7 (29)	*All HPV Neg./PD-L1 Neg.* 3/8 (38)	

**Figure 7:**
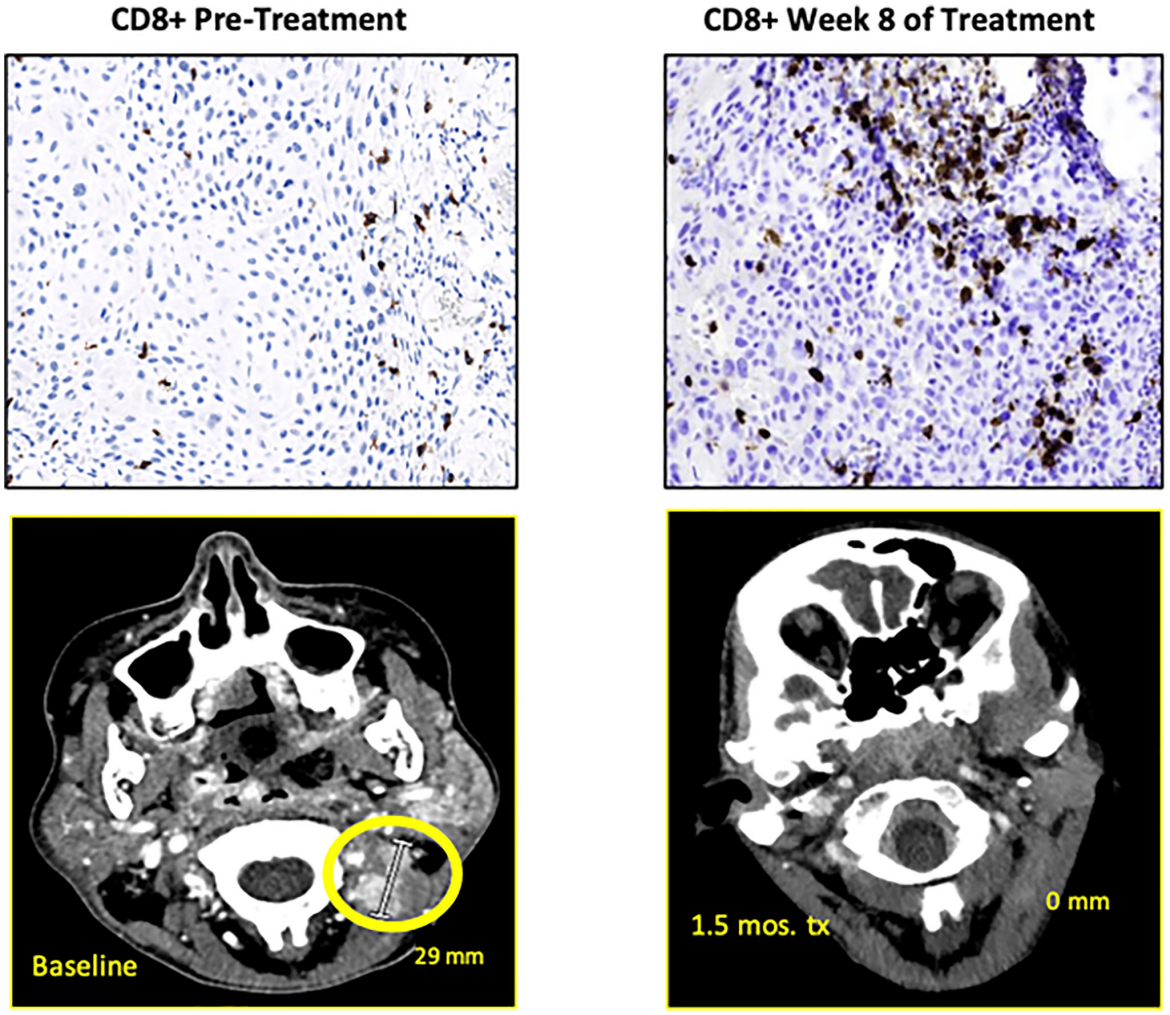
Baseline and on-therapy imaging and biopsy results showing radiographic resolution of tumor and increase of intratumoral COB+ cells.

CD3 and CD8 T-cell tumor infiltration was measured on paired samples from baseline and on therapy around week 8 (after 2 cycles) in 13 (52%) of 25 patients. On therapy tissue was not evaluable in 4 cases. Data on the remaining 9 cases is shown (Table 6). Three (23%) of 13 patients had no malignancy in week 8 biopsy consistent with pathologic complete remission (pCR) (Table 6). Of the 6 patients with paired samples, 4 had RECIST-defined response, and all had significant immune cell infiltration (CD3 and CD8) into the tumor. They were all EphrinB2 positive. Among the three PD-L1 positive cases, two were also EphrinB2 positive, both had increase in T cell infiltration and both had response, while the single EphrinB2 negative/PD-L1 positive case did not show a significant intratumoral immune cell increase and was not a responder. Lastly, there was one case which was EphrinB2 negative and PD-L1 negative, who also did not show significant increase of intratumoral immune cells, and was not a responder. A summary of all cases with paired sample analysis, biomarker status and response is shown (Table 6) (Figure 3A, 3B). A representative case who achieved complete remission is shown (Figure 7).

**Table 6:** Patients (*n* = 9) with paired biopsies at baseline and on therapy, and change in intratumoral CD3 and CD8 T cells

Patient No.	HPV status	RECIST response	EphrinB2 expression	PD-L1 expression	Intratumoral immune cell increase or pCR
1	Positive	SD	Positive	Positive	pCR
2	Positive	SD	Positive	Negative	pCR
3	Positive	SD	Negative	Positive	No change
4	Positive	PD	Negative	Negative	No change
5	Negative	PR	Positive	Positive	Increase
6	Negative	SD	Positive	Positive	pCR
7	Negative	CR	Positive	Positive	Increase
8	Negative	CR	Positive	Negative	Increase
9	Negative	PR	Positive	Negative	Increase

Abbreviations: SD: Stable Disease; PD: progressive disease; PR: partial response; CR: complete response; pCR: pathologic complete response.

## DISCUSSION

This study in relapsed-refractory HNSCC who had progressed after systemic therapy showed favorable activity in the HPV-negative/EphrinB2-positive patients. sEphB4-HSA in combination with pembrolizumab has a safety profile similar to what has been observed previously with no overlapping toxicity. The most common toxicity with sEphB4-HSA is hypertension which requires management.

EphrinB2 expression is higher in HPV-negative patients compared to HPV positive patients (73% vs. 60%). this difference does not account for significant difference in response rates among the two groups. This may be related to the level of EphrinB2 expression. HPV may even suppress EphrinB2 expression as observed in HPV-positive neoplastic keratinocytes *in vitro* [29, 30]. DNA alterations are more frequent in HPV-negative HNSCC which may drive EphrinB2 expression [31]. The median overall survival in the entire cohort of 8.6 months, and 10.9 months in the HPV-negative/EphrinB2 positive group. The HPV-negative population who typically have worse outcomes in HNSCC did better with this combination therapy which should be investigated [7, 11, 12].

Preclinical studies indicate that inhibition of EphrinB2 promotes immune cell recruitment into the tumor [32]. This function is critically required to enhance the efficacy of PD-1-PD-L1 directed therapy. It is known that PD-1 Ab therapy is most effective if the tumors have pre-existing immune cells in the tumor, and PD-1 Ab therapy has low overall response in HNSCC especially in HPV-negative patient population [33, 34]. Increase in recruitment of immune cells when sEphB4-HSA is combined with PD-1 Ab therapy, and a greater than doubling of the overall response rate in the HPV-negative patient population supports complementary and independent functions of each therapy.

Some of the patients developed complete remission on the combination therapy. Genomics and proteomics analysis will help define the patients likely to have rapid tumor regression. In some patients rapid tumor regression seen with this treatment could have undesirable consequences as these patients experienced exposure of underlying tissue and delayed epithelialization of the anatomic defect [35, 36]. Future development for sEphB4-HSA in HNSCC is likely to focus on patients with HPV-negative disease where there is greatest need to improve on the outcomes with pembrolizumab monotherapy.

## MATERIALS AND METHODS

### Patients

This was a phase II, single-arm, non-randomized clinical trial evaluating the efficacy of sEphB4-HSA combined with pembrolizumab (MK-7435) in patients with squamous cell carcinoma of the head and neck (HNSCC). Patients of age ≥18 years old with HNSCC who met eligibility criteria were enrolled. Patients had locally advanced or metastatic disease that had progressed after platinum-based chemotherapy and received up to 2 prior lines of treatment. Patients who refuse first line platinum-based chemotherapy were also eligible. All patients had measurable disease based on RECIST 1.1 (Response Evaluation Criteria in Solid Tumors), an ECOG (Eastern Cooperative Oncology Group) performance status of 0 or 1, and had baseline tumor tissue available for analysis. Signed informed consent was obtained from each study subject prior to enrollment. Serum, plasma, and peripheral blood mononuclear cells were collected at baseline and every 4 cycles. Tumor tissue was collected at week 8 (cycle 2) on therapy when possible. HPV-positivity was determined in patients with oropharyngeal carcinoma using surrogate p16 positivity in baseline tumor tissue. Patients who received prior therapy with anti-PD-1, or anti-PD-L1 agents were excluded. Other exclusions included known active central nervous system (CNS) metastases, autoimmune disease, pneumonitis, HIV, bleeding disorders, those receiving anti-coagulation or anti-platelet therapy, those with undiagnosed or uncontrolled hypertension (>150/90 mmHg), and those with evidence of stroke or myocardial infarction within 6 months prior to study enrollment. Patients with hypertension controlled with medications were eligible.

### Treatment

Patients received sEphB4-HSA 10 mg/kg once per week on days 1, 8 and 15, and pembrolizumab 200 mg by intravenous infusion, once every 3 weeks, day 1 of each 3-week cycle for up to 24 months. Treatment was discontinued for confirmed radiographic disease progression, unacceptable adverse experiences, intercurrent illness, or noncompliance with treatment. Tumor imaging was performed every 6 weeks (every 2 cycles) by computed tomography (CT) scan of the neck, chest, abdomen, and pelvis and assessed according to RECIST.

The National Cancer Institute Common Terminology Criteria for Adverse Events (NCI CTCAE), Version 4.0 was used to grade and record adverse experiences (AEs) and toxicities. Pembrolizumab was withheld for drug-related toxicities and severe or life-threatening AEs. Treatment was permanently discontinued for all grade 4 toxicities, and certain grade 3 toxicities including renal failure, pneumonitis, infusion reaction, and elevated AST, ALT, or bilirubin. Treatment was continued for any grade hypertension while antihypertensive drugs were initiated; in cases of a second occurrence of grade 3 hypertension, treatment with sEphB4-HSA was held until hypertension returned to grade ≤2 at which point treatment could be resumed with dose reduction. Level 1 dose reduction reduced sEphB4-HSA to 5 mg/kg per week and 2.5 mg/kg per week for Level 2. Patients could have a maximum of 2 dose reductions for drug-related toxicity, after which sEphB4-HSA was discontinued.

### Biomarker Analysis

Immunohistochemistry (IHC) was performed on patient tumor samples obtained at baseline and week 8 (cycle 2) on therapy. Biomarkers analyzed included EphrinB2, PD-L1, immune cell markers CD3, and CD8 for both collections. Patient tissue samples at baseline were sent to Caris Life Sciences for comprehensive tumor sequencing and PD-L1 using Monoclonal Rabbit Anti-PD-L1 Clone 28-8 or 22c3 assays. Scoring for PD-L1 on both baseline and on-treatment tissue samples included tumor and immune cell membrane PD-L1 staining, including cytoplasmic staining in the case of the 28-8 assay. Patients were determined to be PD-L1 positive if their tissue sample at baseline demonstrated ≥1% combined positive score (CPS) in the case of the 22c3 assay or >1% tumor proportion score (TPS) in the case of the 28-8 assay. The scoring procedures and staining protocol are described in the instructions of the commercial assay for squamous cell carcinoma of the head and neck. IHC for EphrinB2 and immune markers was performed at the CLIA approved core laboratory and analyzed by an independent pathologist (I.S.) EphrinB2 assay used Rabbit Monoclonal Anti-Ephrin B2 antibody. Scoring and analysis for EphrinB2 positivity at baseline and on-treatment biopsy was based on tumor cell membrane staining for EphrinB2. Patients were determined to be EphrinB2 positive if their tissue sample at baseline demonstrated ≥1% TPS [37]. p16 staining was done in the CLIA certified clinical laboratory as a routine service. IHC of immune markers was performed to assess immune cell infiltration into the tumor. This included staining for CD3 and CD8. Immune marker analysis was performed on baseline and on-therapy collected tumor tissues [38]. Tumor infiltration was defined as an increase of at least 100% in immune cells quantified by running DAB positive cell detection analysis on areas within the tumor boundary using *QuPath* software on CD3 and CD8 stained slides within the tumor from baseline to on-therapy biopsy.

### Trial oversight

The study was designed and conducted as in investigator-initiated trial (IIT). The study was approved by the Institutional Review Board (IRB) of the University of Southern California, registered at ClinicalTrials.gov as NCT03049618. The IRB and the Data Safety and Monitoring Committee (DSMC) provided the safety monitoring of the study. The trial was conducted in accordance with the protocol with Good Clinical Practice Guidelines, and the provisions of the Declaration of Helsinki. All patients provided written informed consent before study enrollment.

### Statistical analysis

Standard descriptive statistics were used to summarize the demographics and baseline clinical characteristics and the results of this trial. The observed overall response rate (ORR) was reported as percentage and its associated 95% confidence interval (CI) was calculated [39]. Overall survival (OS) was calculated from the time of treatment start to date of death, due to any causes. Progression free survival (PFS) was calculated from the time treatment start to death or disease progression observed whichever occurred first; patients who were alive and free of disease progression were censored at the date that their status was last documented. The Kaplan-Meier product limit method was used to display the OS and PFS pattern over time. Median OS and PFS were based on Kaplan-Meier plots; their associated 95% CIs were calculated using Greenwood’s standard errors formula [40]. Log-rank test was used for testing the association of HPV, P16, EphrinB2, and PD-L1 status with OS and PFS. Fisher’s exact test [41] was used for testing the association of biomarkers with response. All reported *p* values were two-sided and *p* values < 0.05 were considered statistically significant.

## Abbreviations

HNSCC: Head neck squamous cell carcinoma
OS: overall survival
PFS: progression-free survival
ORR: overall response rate
sEphB4-HSA: soluble EphB4-human serum albumin
CR: complete response
PR: partial response
SD: stable disease
PD: progressive disease
HPV: human papillomavirus
